# Current Concepts in Paediatric Femoral Shaft Fractures

**DOI:** 10.2174/1874325001711010353

**Published:** 2017-04-28

**Authors:** Rakesh John, Siddhartha Sharma, Gopinathan Nirmal Raj, Jujhar Singh, Varsha C., Arjun RHH, Ankit Khurana

**Affiliations:** 1 Department of Orthopaedics, Post Graduate Institute of Medical Education and Research, Chandigarh, India; 2 Department of Paediatrics, Indraprastha Apollo Hospital, New Delhi, India

**Keywords:** Children, Elastic nailing, Femur, Fractures, Hip spica, Submuscular plating

## Abstract

Pediatric femoral shaft fractures account for less than 2% of all fractures in children. However, these are the most common pediatric fractures necessitating hospitalization and are associated with prolonged hospital stay, prolonged immobilization and impose a significant burden on the healthcare system as well as caregivers. In this paper, the authors present a comprehensive review of epidemiology, aetiology, classification and managemement options of pediatric femoral shaft fractures.

## INTRODUCTION

Pediatric femoral shaft fractures are uncommon, constituting less than 2% of all fractures in children; yet they are a significant burden on healthcare systems and families as they are the most common fractures requiring hospitalization in children [1, 2]. These injuries often require prolonged immobilisation or surgery [1]. This review will look at the epidemiology, classification and current concepts in the management of pediatric femoral shaft fractures.

## EPIDEMIOLOGY

Pediatric shaft femur fractures are 2.6 times more common in boys than in girls [3-5]. A bimodal distribution has been noted, with the first peak occurring in the age group of 1-3 years (usually low energy) and the second peak during early adolescence period (high energy), which constitutes the majority of the fractures [3, 5]. Although, the etiology of the fracture varies with the age of the child, the most common cause of femur shaft fractures in children is fall from height and road traffic accidents [4, 6]. However, one should keep the suspicion of child abuse in mind while dealing with these fractures in young children as it has been observed that up to 80% of femur shaft fractures occurring in children before walking age are due to abuse [7, 8]. Coffey *et al.* [9] reported that 67% of lower limb fractures in children less than 18 months of age were as a result of child abuse. In children more than 3 years of age, abuse is unlikely to cause femur fractures since bone at this age is significantly stronger in resisting both torque forces and direct blows [4, 6].

Stress fractures of the femoral shaft and neck, although uncommon, are increasingly being noticed in adolescent athletes participating in soccer, basketball, athletics *etc.* and account for 4% of all stress fractures in children [10-12]. A high index of suspicion is needed to diagnose undisplaced stress fractures in order to prevent their eventual progression to a displaced fracture [10-12].

## CLASSIFICATION

There is no universal classification system available for pediatric shaft femur fractures. Fractures are usually classified descriptively either on the basis of i) configuration - transverse/spiral/oblique ii) comminution - comminuted or non-comminuted and iii) presence/absence of soft tissue coverage around fracture- open/closed. The most common type of fracture is a simple, transverse, non-comminuted, diaphyseal fracture accounting for more than 50% of the cases [1].

According to the AO (Arbeitsgemeineschaft fur Osteosynthesefragen) paediatric comprehensive classification of long bone fractures, shaft femur fractures are classified as category 32-D [13]. Sub-categories 32- D 4.1 (complete transverse with an obliquity of 30º or less) and 32-D 5.1 (complete oblique or spiral more than 30^0^) are simple fractures whereas subcategories 32-D 4.2 (multi-fragmentary transverse 30^0^ or less) and 32-D 5.2 (multi-fragmentary oblique or spiral more than 30^0^) are unstable patterns [13].

## MANAGEMENT

Pediatric femur shaft fractures tend to unite rapidly and have a tremendous remodelling potential. Consequently, a wide range of deformity of the initial healed bone is considered acceptable. The acceptable angulation in the coronal and sagittal planes varies from 30^0^ at birth to 15^0^ at 10 years. Rotational malalignment does not remodel and deformity more than 10^0^ in the axial plane is not acceptable [14]. Limb shortening of up to 15 mm can be compensated in children up to 12 years of age by growth acceleration [15].

The decision to manage a femoral shaft fracture by conservative or operative means is affected by a wide number of variables, which include age and weight of patient, the type of fracture, associated injuries/polytrauma and socioeconomic status of the family [16].

Age is the main predictor of the treatment (Table **1**). Fractures in children below 6 years of age are usually managed non-operatively due to the excellent remodelling potential of this age group. Various conservative modalities used include Pavlik harness, traction (Bryant’s traction, skin traction and skeletal traction), hip spica and functional bracing. Immobilization in a Pavlik harness works well for infants up to 6 months of age whereas hip spica is preferred for older children [16].

The treatment of fractures in the age group 5-16 years is controversial with multiple options available and no clear consensus on the preferred modality of management. Surgical options are external fixation, plating (conventional and submuscular bridge plating) and intramedullary nails which can be by flexible nails (titanium nail, Enders nail) or rigid nails [16]. The different treatment options are discussed at length.

## CONSERVATIVE MANAGEMENT

### i) Pavlik Harness

This orthosis is ideal for birth injuries with proximal or mid-shaft fractures in infants.^1^Both stable and unstable fractures can be managed using a Pavlik Harness and a wrap on around the thigh. The proximal fragment lies in flexion due to the pull of the iliopsoas; when the Pavlik harness is applied in moderate flexion and abduction, the distal fragment automatically aligns itself to the proximal fragment [1].

The use of Pavlik harness in infants with femur fractures was popularised by Stannard *et al.* [17] who noted acceptable alignment in all patients with less than 1cm shortening. Podezwa *et al.* [18] compared hip spica application to Pavlik harness application in children less than 1 year of age in a retrospective study. There was no difference in the radiological outcomes in both the groups. They noted that infants treated with spica cast had lower pain scores compared to those treated with Pavlik harness; however, one-third of the patients on hip spicas had skin complications whereas none of those treated with Pavlik harness reported skin complications. Based on the observations of Podezwa *et al.* [18], Pavlik harness application is now the recommended treatment for shaft femur fractures in children less than 1 year of age.

### ii) Hip Spica Casting (Table **2**)

In children 1-6 years of age, hip spica casting has traditionally been the treatment of choice unless there are associated injuries, excessive shortening (>2cm), skin complications which can preclude application of hip spica [19, 20].

Based on the timing of application of the spica, hip spicas have been arbitrarily classified into “immediate” and “early” spicas. When the spica is applied within minutes of presentation to the clinic, it is called “immediate spica casting” whereas when the spica is applied a few days after the injury, it is termed as “early spica casting”.

The ideal position for hip spica application has always been controversial. Numerous studies have reported excellent outcomes for ‘one and a half’ hip spica casting in the 90-90 position/ “sitting spica cast” (i.e both hips and knees in 90^0^ of flexion) [21, 22]. Casting in the 90/90 position permits the child to be placed on a chair or to be easily carried in the lap of the parents and also obliterates the need of bedpans for toiletries [1]. It has also been observed that knee flexion greater than 60^0^ improves maintenance of length and reduction [23]. However, Frick *et al.* [24] observed that excessive traction with increased knee flexion increases the chances of compartment syndrome and skin sloughing. The position of the knee (lesser than 90^0^ flexion), lesser traction and cast padding are critical to avoid this complication.^1^ According to a recent Cochrane review, the safe and effective position is 30^0^ of abduction, 30^0^ to 40^0^ of flexion and external rotation at the hip [25]. The fracture location may also the dictate the amount of flexion needed at hip with more proximally located fractures needing more amount of flexion [20]. All patients should be observed for 24 hours after hip spica application to rule out neurovascular compromise and compartment syndrome [1].

Advantages of spica are excellent union rates, low cost, good safety profile, reduced need of specialised surgical instrumentation/tools and low rate of complications like limb length discrepancy, non-union *etc* [26, 27]. The complications associated with spica casting are maintenance of personal hygiene, transportation difficulties and intolerance of the child to cast [28]. It has been noted that the negative impact of spica casting is more on school going children (i.e. children more than 5 years of age) than in pre-school children [29].

### iii) Walking Spica/Single Leg Hip Spica

Of late, there has been a renewed interest in the concept of “walking spica” or “single-leg hip spica” for selected indications. The walking spica is ideal in stable, low energy fractures in toddlers [30, 31]. It is applied with the ipsilateral knee in 45^0^ of flexion and the hip in 45^0^ of flexion and 15^0^ of external rotation. The hip should be extensively reinforced anteriorly to avoid breakage. Additional advantages offered by walking spica over conventional hip spica are improved function and care and reduced chances of spica syndrome [30-35]. The disadvantage is the increased chance of loss of reduction compared to conventional spica [30, 32].

Epps *et al.* [32] reported that 90% children pulled to stand and 62% children walked independently by the end of the treatment with minimal complications. However, Flynn *et al.* in a comparative study noted that outcomes of children treated with walking and conventional spica were similar [30].

Leu *et al.* [31] too did not find a significant difference in radiological and functional outcomes between single leg and double leg spica casts at cast removal (mean 44 days in both groups). They observed that single-leg casts afforded more comfort during sitting and greater ease on leaving the family home. Fewer caregivers needed to take time off work in the single-cast group and for lesser time duration.

### iv) Traction Followed by Casting/ Functional Orthosis

The eponymous Bryant’s traction was introduced by Bryant in 1873, wherein vertical overhead traction was applied with both hips in 90^o^ flexion and knees in full extension [36, 37]. Due to ensuing vascular complications in few patients, this method went out of vogue [38]. Modified Bryant’s traction was described by Ferry *et al.*, [39] in 1966; knees were placed in 45^o^ of flexion, which reduced vascular complications.

Application of traction before spica casting/functional bracing is indicated in length unstable fractures provided the family members agree to the long period of immobilisation.^1^ The Thompson’s telescope test can be used to detect length unstable fractures [40]. At the time of hip spica application, if > 3 cm shortening is demonstrable with gentle axial compression under fluoroscopic imaging, then the fracture is length unstable and traction should be used [40]. Both skin and skeletal traction can be applied, depending on the weight of the child. Skeletal traction is ideally used when > 5 lb of traction is needed [41]. The preferred choice of site for application of traction pin is the distal femur as proximal tibial pins have been known to cause recurvatum deformity subsequent to proximal tibial physeal plate damage and may also aggravate associated menisco-ligamentous injuries of the knee by over-distracting the knee joint [41-43].

## SURGICAL MANAGEMENT

### i) External Fixation (Table **3**)

**Fig. (1):**
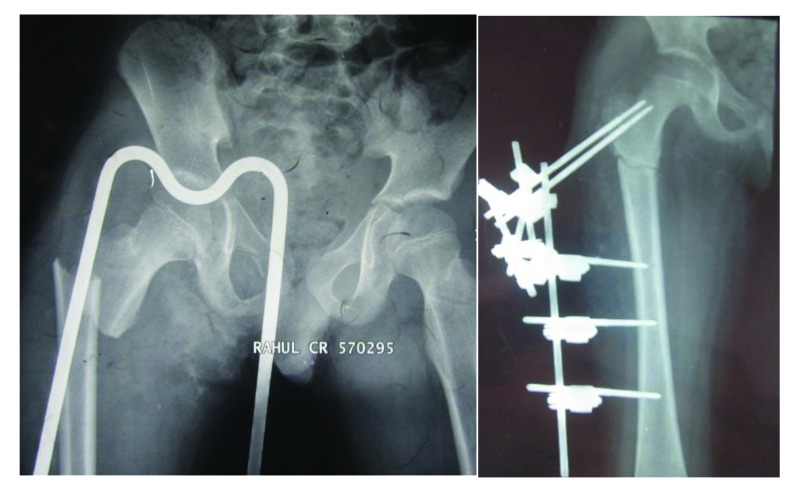
Preoperative and post operative images of an open subtrochanteric femur fracture in a 7 year old male managed by external fixator.

It is the recommended mode of treatment when fracture femur is associated with severe soft tissue injury, head injury and/or polytrauma (damage control orthopaedics) or when the fracture is pathological in nature (Fig. **1**) [1, 44].

External fixator application is associated with complications manifold [45-48]. The main complication is pin tract infection which can occur in up to 72% of patients [45]. Other complications are secondary fractures after implant removal with an incidence ranging from 1% to 22% [45-49]. This means that the fixator should be left in situ for a long period of time until bridging callus is seen in atleast 3 cortices in 2 orthogonal radiological views. They are difficult to use in proximal/distal fractures due to difficulty in placement in the physeal regions. Hip or knee joint stiffness may develop when major soft tissue injuries are present [46].

Fixator removal is usually done after 3-4 months when bridging callus is noted in at least 3 of the 4 cortices on AP and lateral views. An alternative strategy (“portable traction”) is to remove fixator at around 6-8 weeks when early callus is noted and to apply a walking spica. This method minimises stress shielding and allows pin tracts to ossify with the spica acting as a protective splint [1].

### ii) Intra-Medullary Nailing

#### Elastic Stable Intramedullary Nails (Esin) (Table **4**)

Developed by the Nancy group in France [58, 59] in the early 1980’s, this is the most popular method of fracture fixation in the age group of 5-11 years as fractures tend to angulate and/or shorten with spicas due to the bulkier frame. Flexible intramedullary nails are load sharing devices which offer good fixation (relative stability and subsequent fracture union by indirect bone healing/callus formation), are relatively cheaper and have a short learning curve as it is relatively easier to insert and remove these nails [60]. Bone growth is affected minimally, as the need to cross physis can be avoided with these nails; the mean femur overgrowth is 1.2 mm. Operating time and blood loss is significantly reduced [60, 61].

**Fig. (2):**
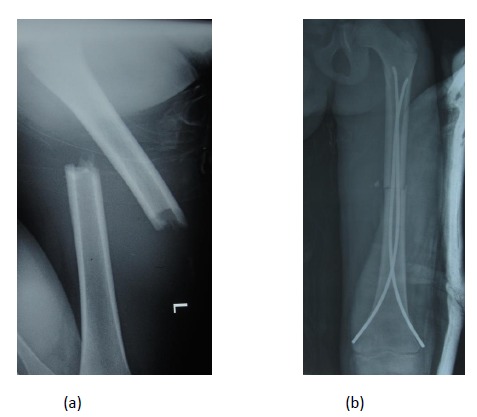
Preoperative and postoperative images of a femoral shaft fracture in a 6 year old girl treated by retrograde elastic stable intramedullary nailing.

The preferred technique for insertion of these nails is a retrograde technique with 2 small incisions (medial and lateral) just above the distal femoral physis (Fig. **2**) [1]. Antegrade nailing from the subtrochanteric area avoids post-operative knee complications [62]. The diameter of the nail should be two-fifths of the diameter of the medullary canal and should be calculated preoperatively [1]. During insertion, it is important to prebend the nails so that the apex of the bend lies across the fracture site [59, 60, 63]. The elastic deformation of the pre-bent nail in a straight medullary canal imparts a bending moment which tends to angulate the fracture. The insertion of another nail of the same diameter from the opposite side balances this moment leading to good stability against bending and torsional forces. This principle is referred to as ‘trifocal buttressing’ [59, 60, 63]. Frick *et al.* [64] observed that retrograde double C configuration is better than antegrade C or S pattern as it offers greater resistance to torsional forces.

Complications include excessive shortening which leads to nail protrusion and limb length discrepancy. The most common complication is pain or skin irritation at the nail insertion site caused by a prominent nail end [63, 66]. Higher rate of unplanned surgeries and malunions have been observed in length unstable fractures and heavy (>50 kg) children [63, 65-67].

• **Antegrade vs retrograde insertion of flexible nails**

Frick *et al.* [64] conducted a biomechanical study to evaluate the stability of simulated transverse and comminuted femoral fractures after retrograde and antegrade flexible nail insertion in five synthetic adolescent-sized femoral bone models each respectively. They noted that retrograde nail fixation demonstrated significantly less axial range of motion and greater torsional stiffness than antegrade fixation in both fracture patterns. However, antegrade nails demonstrated greater resistance to shortening [64].

Mehlman *et al.* [68] conducted a mechanical study to determine whether the stability of ESIN constructs differ in terms of antegrade versus retrograde insertion for the fixation of pediatric distal-third transverse femoral-shaft fractures in 10 synthetic composite adolescent-sized femur models. All the specimens were subjected to 4-point bending followed by axial torsion. They observed that flexural stiffness was significantly greater in the retrograde group (350±72 N/mm) compared with antegrade (195±95 N/mm; p = 0.02). Although antegrade nail insertion is recommended for distal-third femur fractures, Mehlman *et al.* demonstrated that given satisfactory cortical starting points in the distal fragment, retrograde insertion provides greater stability [68].

• **Steel vs titanium flexible nails**

Wall *et al.* [69] compared stainless steel to titanium elastic nails and found that the cheaper stainless steel nails were superior to titanium nails owing to a lesser rate of malunion (6.3% vs. 23.2%). However, in an experimental study by Perez *et al.* [70] it was noted that stainless steel nails were associated with increased gap closure and nail slippage; titanium nails, on the other hand, offered greater stability. It was also observed that stainless steel nails hamper re-modelling and consequently increased the chances of re-fracture [71].

• **Flexible interlocked nailing**

Linhart and Roposch [71] described a method of “locking” flexible Enders rods to maintain leg length and alignment without compromising early postoperative mobility. Cramer *et al.* [72] reported a different locking technique and reported no clinically significant malunions, motion loss, or leg length discrepancy. They recommended Enders nail over TENS system due to the locking capability of Enders nails although they are limited by the canal size [73].

Ellis *et al.* [73] conducted a retrospective review to study locked versus unlocked Ender’s nails in length unstable femur shaft fractures (defined as either a comminuted fracture or a spiral fracture longer than twice the diameter of the femoral shaft). They identified a total of 107 length unstable fractures fixed with Enders nails, of which 37 cases had both Enders rods “locked” through the eyelet in the distal femur with a 2.7mm fully threaded cortical screw. They observed that shortening of the femur and nail migration at 1-6 weeks post-operatively was significantly greater in the non-locked nails group. Also, there were significantly more clinical complaints in non-locked group including limp, clinical shortening, and painful, palpable rods. Based on these observations, they concluded that locked Enders rods are an excellent option to prevent shortening in length unstable fractures and result in no additional complications or added surgical time or increased blood loss [73].

#### Rigid Intramedullary Nails (Table **5**)

**Fig. (3):**
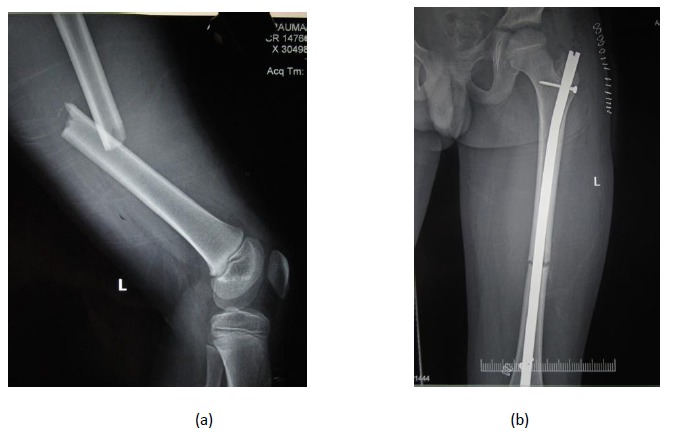
Pre-operative and post-operative radiographs of an 11 year old male with femoral shaft fracture treated by a trochanteric entry rigid intramedullary nail.

Rigid intramedullary nails initially fell out of favour compared to flexible intramedullary nails as these nails had a piriformis entry and hence were associated with avascular necrosis (AVN) of the femur head and with injuries to the growth plate leading to growth arrest [85]. However, with the introduction of trochanteric entry nails (Fig. **3**), which has reduced the chances of osteonecrosis, the use of rigid intramedullary nails in adolescents is on the rise again [86, 87].

Current literature suggests that rigid intramedullary nail with a trochanteric entry point is the preferred mode of fixation of shaft femur fractures in adolescents [1]. However, growth disturbance due to physeal plate damage is still a concern with these nails and hence it is not preferred for use in children less than 12 years.

### iii) Plate Fixation

Pediatric trauma surgeons have largely moved away from the traditional open reduction and compression plating to the more modern submuscular bridge plating which offers stability without disturbing the vascularity of the fracture fragments hence leading to early union.

### SUBMUSCULAR BRIDGE PLATING (Table **6**)

**Fig. (4):**
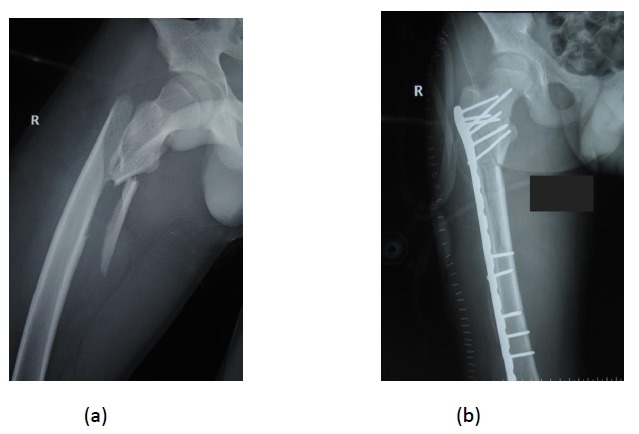
Preoperative and post operative radiographs of a comminuted subtrochanteric fracture with extension into the femoral shaft managed by submuscular bridge plating.

Submuscular bridge plating provides excellent stability; it is especially useful in the management of proximal/distal fractures that are not suitable for IM nailing/external fixation (Fig. **4**) [93, 94]. This method can also be used in pathologic fractures and associated head injuries. Disadvantages are the difficulty in implant removal due to cold welding of locking screws to the plate, significant blood loss and relatively higher learning curve [93-100].

## CONCLUSION

Femoral shaft fractures in children are amongst the commonest fractures necessitating hospitalization. The major determinant of treatment modality is age of the child. Fractures in children below 6 years of age can be managed non-operatively with excellent outcomes. Elastic stable intramedullary nails are preferred for children < 11 years of age or those with body weight < 50 kg with a length stable transverse or short oblique fracture. Length unstable fractures and fractures at the proximal ends of femur may be managed by submuscular plating or external fixation. For children above 11 years of age or those with body weight > 50 kg, rigid intramedullary nailing or submuscular plating is preferred. Piriformis fossa entry nails should be avoided to prevent the complication of avascular necrosis of femoral head.

## CONFLICT OF INTEREST

The authors confirm that this article content has no conflicts of interest.

## Figures and Tables

**Table 1:** Summary of suitable treatment options available for management of pediatric shaft femur fractures according to age of the child.

**Age of child**	**Preferred management**	**Other modalities**
**0-6 months**	Pavlik harness	Hip spica
**6 months to 2 years**	Hip spica	Traction followed by spica
**3-5 years**	Hip spica	Traction followed by spica/orthosisExternal fixation (Rare)Flexible intramedullary nails (Rare)
**6-11 years**	Flexible intramedullary nails	Traction followed by spicaExternal fixationSubmuscular plating
**More than 12 years**	Rigid intramedullary nails (Trochanteric entry)	Flexible intramedullary nailsExternal fixationSubmuscular plating

**Table 2:** Summary of selected major studies on hip spica application in the management of pediatric femur shaft fractures.

**Authors**	**Year**	**Study design**	**No. of fractures**	**Treatment**	**Results & complications**	**Remarks**
**Cassinelli *et al.* [33]**	2005	Retrospective review	145	Immediate spica	Acceptable alignment in all patients.Low complication rate	Immediate spica is a safe procedure
**Illgen *et al.* [23]**	1998	Retrospective review	114	Early spica	Successful in 86% patients	Procedure of choice <6 years
**Czertak and Hennrikus *et al.* [34]**	1999	Retrospective study	23	Early Spica	Average no. of days in cast 42Mean shortening at cast removal 1 cm	Procedure of choice <6 years
**Epps *et al.* [32]**	2006	Retrospective review	45	Single-leg spica cast	Failures (2)Repeat casting (2)Rotational malunion (1)No radiographic malunions	Recommended in low energy fractures in young children
**Flynn *et al.* [30]**	2011	Prospective study	45	Traditional spica v/s “walking” spica	Similar outcomes in bothMore chances of wedge readjustment in walking spica	Less burden of care on family in walking spica
**Leu *et al.* [31]**	2012	RCT	52	Single leg spica v/s double leg spica	Similar functional and radiological outcomes in both groups.Single-leg spica group was more likely to fit into car seats and chairs comfortably.Caregivers took less time off work.	Single leg spica is effective and safe.

**Table 3:** Summary of selected major studies on external fixator (EF) application in the management of pediatric femur shaft fractures.

**Authors**	**Year**	**Study design**	**No. of fractures**	**Treatment**	**Results & complications**	**Remarks**
**Aronson *et al.* [50]**	1992	Retrospective review	44	Primary external fixation	10% re-application or casting8.5% pin tract infection	Recommended Primary EF use.
**Matzkin *et al.* [51]**	2006	Retrospective review	40	External fixation f/b dynamization	Refractures rate 2.5%100% union rate in those with cortical contact (25)72.5% EF dynamized prior to EF removal	Pin tract infections common (52.5%)
**Bar-on *et al.* [52]**	1997	RCT	20	External fixation v/s flexible nails	Early post-op course similarMore callus, faster union, shorter recovery time, better muscle strength in nailing group	EF recommended only for open/severely comminuted fracturesFlexible nail use recommended
**Kapukaya *et al.* [53]**	1998	Retrospective review	57	EF in closed femur fractures	Low complication ratesPin tract infection (3)Refractures (1)	Recommended EF use
**Davis *et al.* [54]**	1995	Retrospective review	15	Orthofix EF	100% fracture unionPin tract infection (5)Refractures (1)	Recommended EF use
**Hedin *et al.* [48]**	2004	Prospective study	98	External fixator	59 cases of LLD, 35 pin tract infections and 2 re-fractures	Recommended EF use
**Sola *et al.* [55]**	1999	Retrospective study	39	Orthofix EF	Auxiliary pin used in 16 cases	Use of auxiliary pin reduced malunion and re-manipulation rates.
**Domb *et al.* [56]**	2002	RCT	53	Static v/s dynamic EF	Similar results in both groups	No effect of dynamization on union time and complication rate
**Gregory *et al.* [47]**	1996	Retrospective study	27	EF	8 major complications in 6 patients29 minor complications in 20 patients	Careful attention to operative technique and post-operative care needed
**Barlas *et al.* [57]**	2006	Prospective study	40	EF v/s flexible IM nails	More complications with EF:Pain (3)LLD (2)Malalignment (4)No complications in nailing group	Flexible nail use recommended.EF recommended only for open/severely comminuted fractures.

**Table 4:** Summary of selected major studies on ESIN in the management of pediatric femur shaft fractures.

**Authors**	**Year**	**Study design**	**Mean age**	**No. of patients**	**Treatment**	**Results & complications**	**Remarks**
**Flynn *et al.* [60]**	2001	Prospective Review	10.2	58	ESIN	Excellent/ satisfactory outcome in 57 of the 58 cases	TENS may be an ideal implant to stabilize paediatric femur fractures.
**Aktekin *et al.* [74]**	2007	Prospective Study	9.6	21	ESIN	Mean time to union 13 weeksNo malunion.	ESIN is treatment of choice in 6-12 year age group.
**Carey *et al.* [75]**	1996	Retrospective review	12.5	25	Antegrade flexible nails	No non-union/malunion	Treatment of choice in 6-12 years age group
**Singh *et al.* [76]**	2006	Prospective study	11.26	35	ESIN(retrograde)	100% union rateMean time to union 9.6 weeks.	Ideal implant for pediatric femur fractures
**Li Y *et al.* [77]**	2008	Experimental study	-	-	ESIN	Obese children undergoing stabilization of mid-shaft femur fracture with TENS are at risk for loss of reduction.	-
**Saikia *et al.* [78]**	2007	Prospective study	10.8	22	ESIN	100% union rateMean time to union 8.7 weeks	Ideal implant for pediatric femur fractures
**Narayana *et al.* [79]**	2004	Retrospective study	13.7	78	ESIN	Proper nail advancement and fracture comminution are important factors regarding complications of ESIN	Ideal implant for pediatric femur fractures.Most complications are minor.
**Sagan *et al.* [80]**	2010	Retrospective review	10.7	70	ESIN	Anterior bowing greater than 15 degrees is the most common malunion noted with TENS.	Bowing may be reduced if at least 1 of the nails is inserted with the tip pointing in an anterior direction
**Luhmann *et al.* [66]**	2003	Prospective study	6	39	ESIN	Technical pitfalls with TENS can be minimized by leaving less than 2.5 cm of nail out of the femur and by using the largest nail sizes possible	Outcomes were associated with the patient's weight and size of the nails implanted
**Reynolds *et al.* [81]**	2012	Retrospective cohort study	12.6	22	AdolescentLateral femoral (ALFN) nailVs. ESIN	Older, heavier pediatric patients treatedwith ALFNs had a shorter recovery time compared to ESIN group.	Meantime to full weight-bearing significantly less for theALFN group.However, theoutcomes for both groups were satisfactory
**Houshian *et al.* [82]**	2004	Prospective review	6	31	ESIN	All fractures united at a median of 7 weeks. LLD was up to 1 cm in 6 children.	ESIN is a safe method for the treatment of femoral shaft fractures in children between 4-11 years
**Anastasopoulos *et al.* [83]**	2010	Retrospective study	10.3	36	ESIN	50% children had a LLD without functional disability.No clinical mal-alignment observed.	Flexible nailing of diaphyseal fractures of the femur is a reliable method; small learning curve; allows early mobilisation
**Sink *et al.* [84]**	2005	Retrospective review	8.9	39	ESIN (stable V/s unstable fracture pattern)	62% complications recorded. 8 patients (21%) underwent unplanned surgery prior to complete fracture union	“Length-Unstable” femur fractures require methods of treatment other than TENS

**Table 5:** Summary of selected major studies on rigid intramedullary interlocking nails in the management of pediatric femur shaft fractures.

**Authors**	**Year**	**Study design**	**Mean age/Range**	**No. of patients**	**Treatment**	**Results and complications**	**Remarks**
**Reeves *et al.* [88]**	1990	Retrospective Study	13.9	90	Traction + cast (41 patients) v/s Intramedullary nailing (49 patients)	The operative group had a mean hospital stay of 9 days vs 26 days for non-operative group and had fewer complications.	IM fixation better than conservative management
**Kirby *et al.* [89]**	1981	Retrospective Study	11.6	25	(Traction + cast) v/s Intramedullary nailing		IM fixation better than conservative management
**Beaty *et al.* [85]**	1994	Prospective Study	10-15	30	IM nail	100% fracture union. 1 case of asymptomatic AVN of femur head	IM nail reasonable alternative for the treatment of isolated femur shaft fractures in adolescents with polytrauma.
**Momberger *et al.* [90]**	2000	Prospective cohort study	10-16	48	IM nail	All fractures united. No significant deformity/shortening/malunions/ AVN.	IM nailing through trochanteric point is safe & effective for treating femur fractures in adolescents.
**Kanellopoulos *et al.* [91]**	2006	Prospective Study	11-16	20	IM nail	No major complications. All fractures healed within 9 weeks and patients returned to pre-injury activity level.	Excellent results with good surgical technique involving GT entry point.
**Townsend & Hoffinger *et al.* [92]**	2000	Retrospective Study	12-17	34	IM nail	No patient had AVN of the femoral head or other major complications.	The trochanteric tip entry point is recommended for IM nailing of femoral shaft fractures in children and adolescents.

**Table 6:** Summary of selected major studies on submuscular bridge plating in the management of pediatric femur shaft fractures.

**Authors**	**Year**	**Study design**	**Mean age**	**No. of patients**	**Treatment**	**Results & complications**	**Remarks**
**Eidelman *et al.* [95]**	2010	Retrospective Review	8-16	11	Submuscular plating	All fractures united in proper alignment without deformity. 1 patient had 2 cm shortening. No complication related to hardware failure	Submuscular platingof adolescent femoral fracture with precontoured plate is effective.
**Sink *et al.* [96]**	2006	Retrospective study		27	Submuscular Bridge Plating	100% union rate. No intraoperative/postoperative complications	Reasonable option for operative stabilization of comminuted and unstable fractures.
**Agus *et al.* [98]**	2003	Retrospective Study	11.3	14	Submuscular Bridge Plating	Mean healing time 12.4 weeks.Angulation >10^0^ seen in 1 patient.	Bridge plating is effective treatment method for the closed comminuted fractures of the proximal and distal thirds.
**Hammad *et al.* [99]**	2008	Retrospective study	9.4	15	Submuscular Bridge Plating	100% union rate. Screw failure in form of bending or breakage occurred in 2 patients, without clinical consequences. Average femoral lengthening 2.3 mm in 6 patients and 2 mm tibial lengthening in 4 patients.	Reliable method for the treatment of femoral shaft fractures in skeletally immature patients.
**Abdelgawad *et al.* [100]**	2013	Retrospective Review	9	58	Submuscular Bridge Plating	All fractures healed well and all patients returned to full activity. 1 patient had implant failure and other, deep infection in an old open fracture.	Submuscular bridge plating is preferred method for unstable fractures or fractures of the proximal and distal shaft.

## LIST OF ABBREVIATIONS

ALFN: = Adolescent Lateral Femoral Nail
AVN: = Avascular Necrosis
EF: = External Fixator
ESIN: = Elastic Stable Intramedullary Nail
GT: = Greater Trochanter (of femur)
IM: = Intramedullary
